# A Self-Adapting System for the Automated Detection of Inter-Ictal Epileptiform Discharges

**DOI:** 10.1371/journal.pone.0085180

**Published:** 2014-01-15

**Authors:** Shaun S. Lodder, Michel J. A. M. van Putten

**Affiliations:** 1 Clinical Neurophysiology, MIRA-Institute for Biomedical Technology and Technical Medicine, University of Twente, Enschede, The Netherlands; 2 Department of Neurology and Clinical Neurophysiology, Medisch Spectrum Twente, Enschede, The Netherlands; Universiteit Gent, Belgium

## Abstract

**Purpose:**

Scalp EEG remains the standard clinical procedure for the diagnosis of epilepsy. Manual detection of inter-ictal epileptiform discharges (IEDs) is slow and cumbersome, and few automated methods are used to assist in practice. This is mostly due to low sensitivities, high false positive rates, or a lack of trust in the automated method. In this study we aim to find a solution that will make computer assisted detection more efficient than conventional methods, while preserving the detection certainty of a manual search.

**Methods:**

Our solution consists of two phases. First, a detection phase finds all events similar to epileptiform activity by using a large database of template waveforms. Individual template detections are combined to form “IED nominations”, each with a corresponding certainty value based on the reliability of their contributing templates. The second phase uses the ten nominations with highest certainty and presents them to the reviewer one by one for confirmation. Confirmations are used to update certainty values of the remaining nominations, and another iteration is performed where ten nominations with the highest certainty are presented. This continues until the reviewer is satisfied with what has been seen. Reviewer feedback is also used to update template accuracies globally and improve future detections.

**Key Findings:**

Using the described method and fifteen evaluation EEGs (241 IEDs), one third of all inter-ictal events were shown after one iteration, half after two iterations, and 74%, 90%, and 95% after 5, 10 and 15 iterations respectively. Reviewing fifteen iterations for the 20–30 min recordings 1took approximately 5 min.

**Significance:**

The proposed method shows a practical approach for combining automated detection with visual searching for inter-ictal epileptiform activity. Further evaluation is needed to verify its clinical feasibility and measure the added value it presents.

## Introduction

Regardless of all the technological advances in recent years, routine scalp EEG is still used as the standard clinical procedure for diagnosing epilepsy. Not much has changed regarding visual analysis during diagnosis, even though computerized algorithms have been proposed to assist with reviewing (see [1], [2])). The standard diagnostic strategy in a first-time seizure patient is to perform a routine 20–30 min scalp EEG recording and determine if any inter-ictal epileptiform discharges (IEDs) are present. IEDs appear in the raw signal in the form of spikes, sharp waves, poly-spikes, or spike and slow-wave discharges. Given that these patterns are correlated with a high likelihood of a recurrent seizure, their presence play an important role in the diagnosis of epilepsy.

Although the presence of IEDs are very indicative of epilepsy, their absence on the other hand does not exclude the disease. Patients are often required to return for a follow-up recording if the routine EEG is normal. Visual inspection combined with a manual search for IEDs is time consuming and requires an experienced electroencephalographer. Longer recordings and sleep-deprived EEGs have shown to improve the chances of finding inter-ictal activity and thereby yield higher diagnostic efficiency [3]–[6], but given the visual burden already with shorter recordings, this is difficult to implement on a routine basis. Beyond the diagnosis of epilepsy, there is also a need to accurately mark epileptiform activity and investigate properties such as the potential seizure focus and epilepsy type.

Computer-assisted IED detection can help reviewers to find relevant information and reduce the burden of visual analysis. By finding all possible inter-ictal events and presenting them to the reviewer, a page-by-page inspection of the recording can be avoided. Two relevant measures of performance for automated spike detection methods are (*i*) their sensitivity to find IEDs, and (*ii*)the average false detection rate. If the sensitivity is too low, a reviewer cannot count on the results of the automated analysis alone to exclude IEDs from the recording. On the other hand, if the false detection rate of an algorithm is too high, the reviewer will spend more time filtering through detected events than by simply performing a visual review himself.

Many detection algorithms have been proposed and a wide range of techniques have been suggested. Recent studies have focused on either one or a combination of the following approaches: parametric methods; mimetic analysis; Fourier or wavelet analysis; artificial neural networks; template matching; context-based rules; and event clustering [7]–[22]. Clearly, a lot of thought has been given to effective ways of detecting inter-ictal activity. However, almost all of these methods require thresholding to separate IEDs from other events in some way or another, making them limited in achieving both high sensitivities and low false detection rates at the same time. Low thresholds will result in high sensitivities with many false detections, whereas high thresholds will reduce the number of false detections but also lower the sensitivity.

Instead of simply searching for another spike detection technique, this study focused on finding a practical approach to combining automated detection with manual searching. This was achieved by bringing together the detection and review phases with the help of detection certainty values for each event, and updating detected event certainties with the help of user feedback. With our approach, we were able to circumvent the limitation imposed by detection algorithms where a threshold value has to be chosen to achieve either high sensitivities or low false positive rates. By design, the described system learns from the feedback it receives and updates the certainty values of the remaining nominations to prioritize more likely events. In addition, the feedback is also used to update the system on a global level, which aims to improve future detections and reviews.

The ultimate goal of this system is to improve reviewing accuracy and reduce standard review times to below the average of manual detection.

## Methods

This section is divided into two parts. The first part reviews the template-based spike detection algorithm described in [16] which we also use in this study, and summarizes the main steps needed to obtain a collection of detections which serve as “nominated” inter-ictal events. The second part explains how these nominations are grouped together and presented to the reviewer for verification, and how the proposed system can adapt during verification to learn from reviewer feedback. The template-based detection method was chosen for two reasons. First, by using a wide range of IED samples extracted from EEG training data and using the principle of voting and reliability to prioritize detections, the system is able to scale to any recording given that the IEDs it contains are similar in morphology than the templates in the database. Secondly, by using this technique, we were able to assign a certainty value to each detection which enabled us to sort the events in decreasing order of likelihood.

### Template-based IED detection and nominating inter-ictal events

#### Collection and training of a template database

**Figure 1:**
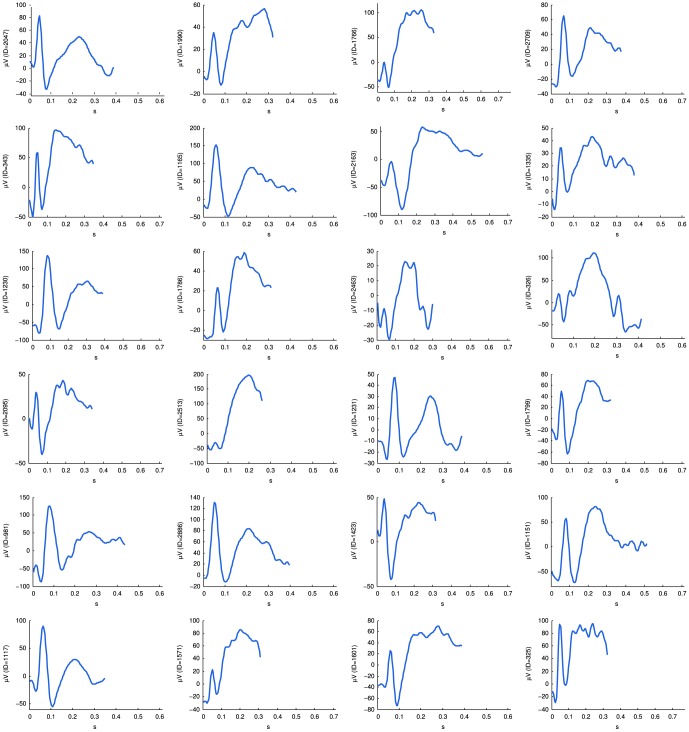
Sample template waveforms collected from a training dataset. To find inter-ictal epileptiform activity in new unseen EEGs, each template searches for matching waveforms with high correlations to themselves. Thereafter, individual template detections are combined to form “IED nominations” which are then presented to the reviewer in decreasing order of likelihood.

To locate possible IEDs in an EEG recording, a database of templates is used to find high correlations with events that represent inter-ictal epileptiform activity. Each template represents a sample IED waveform, and together with this the number of detections it has made in the past are stored including the outcomes of these detections, i.e. true or false positive. The database requires training only once with a training dataset and can thereafter be used on any new EEG to find inter-ictal epileptiform events. For building a database of IED template waveforms, a training set of eight EEGs were used with a total recording length of 175 min. For the training set, an experienced electroencephalographer (MvP) was asked to mark all inter-ictal events in each recording where a clear spike-wave discharge could be seen. Spike-wave events were marked individually on all channels where they were visible. The review took place in three montages: common reference, bi-polar, and Laplace. The training dataset consisted out of 482 inter-ictal events, and from them a total of 2973 spike-wave samples were marked across all channels and montages. Using these marked events as our templates, a database was created where each underlying epoch of data was extracted and stored. Templates ranged from 212 to 860 milliseconds in length. Some sample waveforms are shown in Fig. 1.

After extracting the templates, their ability to find and discriminate between other IEDs from the same or other recordings and non-epileptiform activity was determined. This was done by finding a time-shifted correlation between each template and all EEG channels for all EEGs in the training set with the same montage. Locations were found where the templates had correlations above 0.85, and the underlying EEG segment was extracted to calculate additional properties to further determine the relationship between itself and the template by which it was detected. These properties related to matching variance, amplitude differences, and the ratio between the detected epoch and EEG segments preceding it. For a detailed description of the properties, see [16]. Using the correlations between the detected epochs and the template, together with the additional properties and a known outcome for each detection, a linear support vector machine (SVM) was trained with these parameters as features to learn the difference between true and false detections. The trained SVM for each template together with its score of true and false positives was stored in the database for further use during detection. During the training phase, templates were discarded if they had zero detections, only false detections, or a small number of correct detections and no false detections. After discarding unwanted templates, a total of 2256 remained.

#### IED detection in new EEG recordings

For the detection of inter-ictal activity in new recordings, a similar procedure was used as during learning: for each template in the database, a time-shifted correlation is calculated on every EEG channel in three montages: common reference, bi-polar and Laplace. Epochs with correlations above 0.85 are “nominated” as IED events if they satisfy both the property constraints for that template and it's SVM classifies it as an IED. Each nomination is also assigned a reliability value, which is calculated as 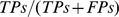, where 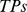 and 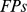 are the total number of true and false predictions that the template has made over all time (note that these scores are saved together with each template in the database).

### Using IED nominations to find inter-ictal events

Our main goal in this study was to combine automated methods with visual reviews to obtain a clinically feasible method for computer-assisted detection of inter-ictal epileptiform discharges. By not discarding any events or using any thresholds during the detection phase, the system was not optimized for achieving a high specificity. Instead, our focus was on providing the reviewer with as many inter-ictal events as possible within a reasonable amount of time. By using an efficient algorithm to present the reviewer with the most probable events first, it is not required of him to review all the detected events in order to draw informed conclusions. Given that the review process is interactive and that no thresholds are used, many of the nominated events will be false positives which are not necessarily presented to the reviewer and ROC curves cannot be used fairly to evaluate the benefits of such a system. Instead, we aim to measure its performance by the percentage of IEDs in a recording that can be shown within a feasible amount of time or minimum number of iterations.

**Figure 2:**
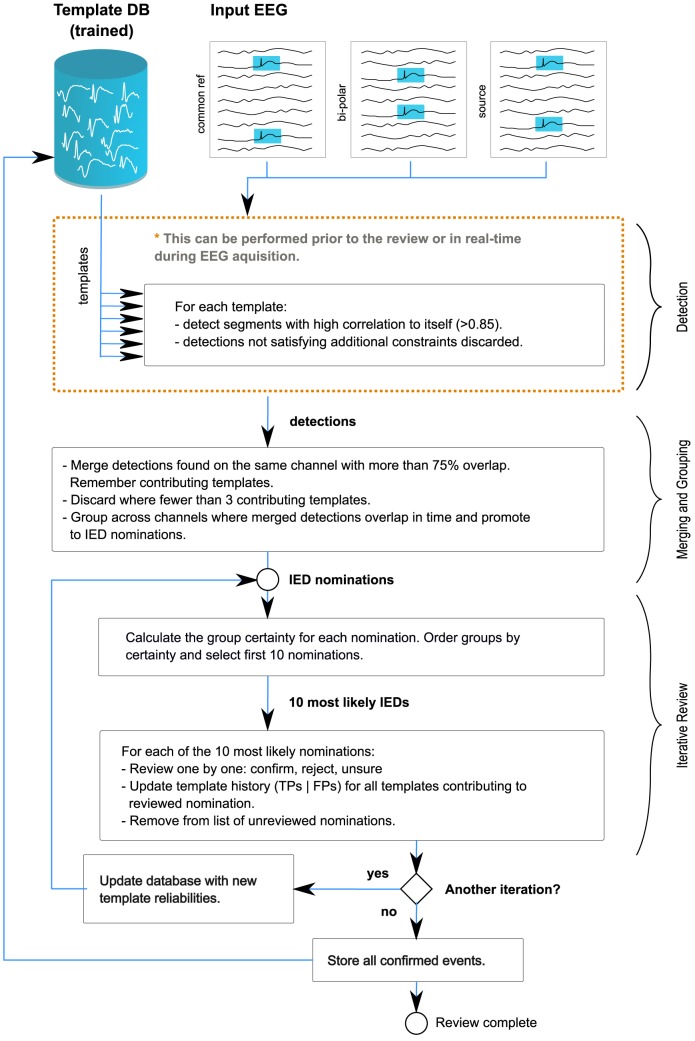
Outline of the IED detection, grouping, and presentation steps. Multiple detections of inter-ictal activity are made using a database of matching template waveforms. Individual template detections are merged and grouped (see Fig. 3) to form IED nominations, and the nominations are presented for review in an iterative manner, ordered by nomination certainty.

To graphically illustrate how the adaptive reviewing method works, a flow diagram of the described system is shown in Fig. 2. As a first step, all IED nominations are found using the template-based IED detection method described in the previous section. As shown in the diagram, the detection phase can be performed independently at any time before the EEG review takes place. Using Fourier optimizations to calculate the correlations (circular convolution), this takes approximately 12–14 min for a 20–30 min recording. This can occur directly after the recording, after an upload to the hospital database, or even in real-time during acquisition. Because very few reviews will occur directly after a routine recording in practice and that this step can be completely automated, it can be considered as part of the acquisition and preprocessing step, and not as part of the actual review time for the electroencephalographer.

#### Grouping

**Figure 3:**
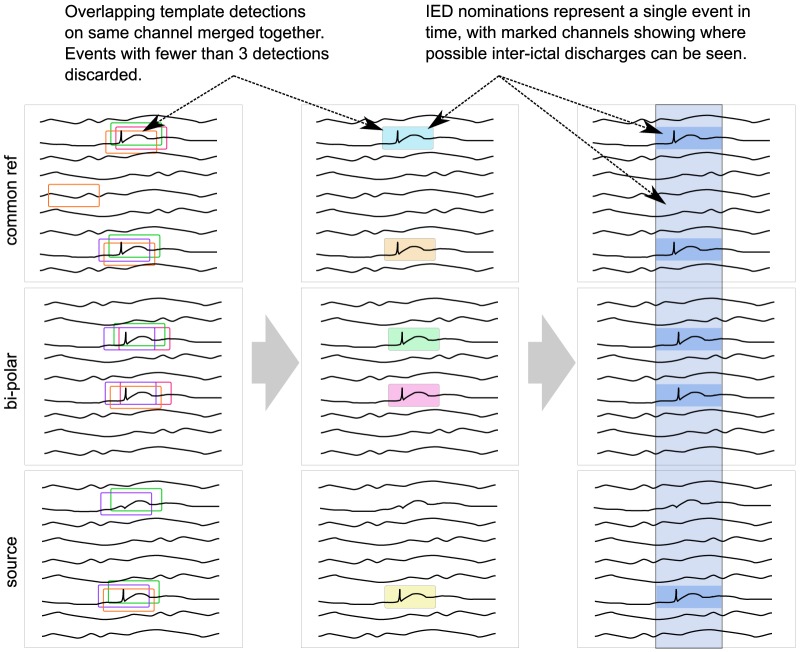
Grouping and merging of template detections. Overlapping templates are merged together and grouped to form single IED nominations, which are presented to the reviewer as a single event in time. Channels containing template detections are highlighted to point out where inter-ictal activity was found.

When the computer-assisted review starts, all IED nominations from individual templates are loaded and overlapping events are merged to form grouped nominations. This is illustrated in Fig. 3. Merging takes place in two steps. First, overlapping template nominations are combined on the same channel if they overlap by more than 75%. Grouped events with fewer than three nominations are discarded. Next, overlapping groups over different channels are merged together where their onsets start within one second from the first item in the group. After these two steps, a number of grouped nominations have been obtained which point to segments in time where possible inter-ictal activity may exist.

#### Group presentation combined with reviewer feedback

If the entire list of group nominations were to be shown to a reviewer, more than 99% of the inter-ictal discharges in each recording will be found (see results [16]). However, this method has the disadvantage of also having a very high false positive rate, and without a smart way of presenting the detections, the reviewer is better off searching for IEDs manually. Fortunately, and what also sets this method apart from other techniques, is that each grouped nomination has an assigned certainty value based on a combination of two factors: (*i*) the correlation of each contributing template to the detected segment, and the reliability of those templates in making accurate detections. The calculation of each nomination group's certainty value is shown in the Appendix. Using the certainty value to the system's advantage, a limited number of nominations with high likelihoods are shown to the reviewer, and in an iterative manner, more can be requested until the reviewer is satisfied with what has been seen. By presenting the nominated events in iterations, the system is able to receive feedback during the review. This allows it to update the reliability of the templates and thereby show more reliable detections first.

Implementation of the described procedure is as follows: Using the entire pool of nominations obtained from the merging and grouping phase, group certainty values are calculated for each nomination. The ten nominations with highest group certainties are then shown to the reviewer one at a time, and the reviewer is given the chance to either confirm the nomination as an IED, reject it, or label it as unsure. After the ten nominations have been graded, their outcomes are returned to the system and the reliabilities of the contributing templates are updated. Given that template reliabilities affect the group certainties of each IED nomination (see Appendix), the group certainties of the remaining nominations are updated. Thereafter, another iteration takes place where ten of the remaining group nominations with highest certainties are shown. This process continues until the reviewer is satisfied with the events seen or until all nominations have been shown. When the review is complete, all confirmed nominations are stored and marked in the recording as detected IEDs.

#### Global update for sustained learning

Apart from making the review of automated detection more efficient by showing the most probable nominations first, this method also allows the system to implement a globally adaptive technique for improving future detections. After an EEG review is complete, the template database is updated to include the additional number of true and false positives made by each template. As described above and also shown in the Appendix, template reliabilities are measured by the number of true and false positives made in the past. Given that more classifications are added over time, a more accurate measure of each template's reliability can be derived. Therefore, over time with continuous use of this technique, reliable templates will become more reliable and have more influence during detection, whereas less reliable templates will receive a lower weight and have less influence.

### Subjects and data

A dataset from the department of Clinical Neurophysiology at the Medisch Spectrum Twente (MST) Hospital in the Netherlands was used to evaluate the proposed method. All EEG data were obtained as part of routine patient care and anonymized before use. Our evaluation dataset consisted of 15 EEGs with a total recording length of 306 min and 241 marked IEDs. The inter-ictal events were marked prior to our evaluation by an experienced electroencephalographer (MvP), and given the time consuming nature of this, only the onset and duration of IEDs were marked and not specific channel information. Table 1 provides additional information about each recording in the evaluation set. Recordings were made using a standard 20–30 minute protocol with the Brainlab EEG system. Ag-AgCl electrode caps were used with electrodes placed according to the 10–20 system, and impedances were kept below 5 kΩ to reduce polarization effects. Recordings were made at a sample rate of either 250 Hz or 256 Hz and band-pass filtered between 0.5–30 Hz. The data was downsampled to 100 Hz to increase the performance of the system, and eye blink artifacts were reduced using an independent component analysis filter.

**Table 1:** Subject information of the EEGs used to test the detection and reviewing method.

Subject	Epilepsy type	Age	Duration
		(years)	(min)
S1	generalized idiopathic	45	16:19
S2	absence	26	20:00
S3	absence	10	21:30
S4	generalized other	50	20:19
S5	generalized other	7	20:40
S6	temporal lobe	51	21:00
S7	generalized other	44	20:00
S8	generalized idiopathic	12	22:30
S9	temporal lobe	17	20:00
S10	generalized other	10	20:00
S11	absence	42	22:19
S12	generalized idiopathic	10	19:30
S13	temporal lobe	7	20:19
S14	generalized idiopathic	4	21:10
S15	generalized other	44	20:00

## Results

**Figure 4:**
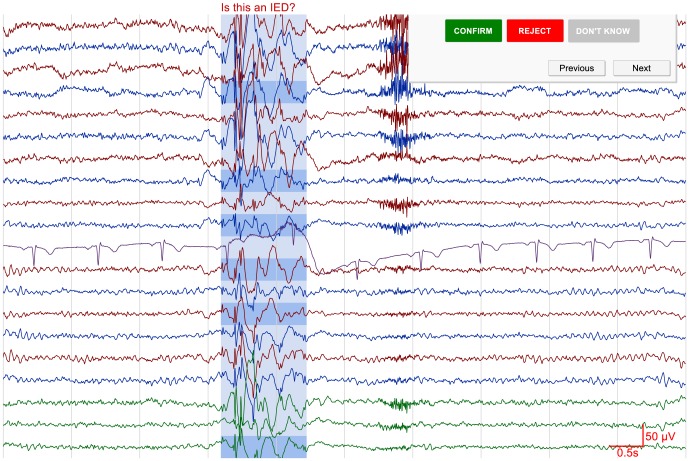
A screenshot showing how the review process works. For each iteration, the reviewer is presented with 10 nominations, one at a time, and is given the opportunity to either confirm, reject, or ignore each event as inter-ictal epileptiform activity (see top right panel). After the ten nominations have been graded, the user can choose to either stop with the review or to review another ten events. Nomination certainties are updated between iterations.

Using the described method, IED nominations were found for all of the 241 IEDs in the evaluation dataset. Given however that no thresholds were used to discard events with low certainties, the system would have a very high false positive rate if all nominations had to be shown to a reviewer. As described in the Methods, iterative reviewing is introduced to help minimize the number of presented false positives. An example of the review process is shown in Fig. 4. A software application is used to show 10 seconds of EEG at a time with the nominated event centered in the middle of the screen. The grouped nomination is highlighted to indicate its onset and duration, and additional highlights are placed on the channels where the templates have found possible IED waveforms. The reviewer is presented with three options in the top-right corner of the screen. From these options the reviewer can decide to either confirm, reject, of ignore the displayed nomination as an IED. The simplicity of this approach allows the user to evaluate nominations in a fast and effective manner. Although only measured during the development of this algorithm, the average duration of visually reviewing one iteration consisting of ten nominations was approximately 20 seconds.

Table 2 shows the number of IEDs detected per recording for each iteration during the review. To provide consistency during the evaluation, nominations were confirmed by comparing them to the marked IEDs obtained earlier from visual inspection. The first and second columns provide the subject ids and number of marked IEDs, and the remaining columns show the number of confirmed IEDs after each iteration of the review. Fifteen iterations were used to evaluate the method with ten nominations presented per iteration. We see that approximately one third of all IEDs are shown within the first iteration of the review and already half of them after two iterations. After five iterations 74% of the events were found, and with ten and fifteen iterations the number of detected IEDs increased to 90% and 95% respectively.

**Table 2:** Adaptive iterative reviewing: IED nominations are iteratively presented to the reviewer one-by-one using 10 events per iteration.

Subject	#IEDs	IEDs confirmed up and until iteration														
		1	2	3	4	5	6	7	8	9	10	11	12	13	14	15
S1	13	8	11	13	13	13	13	13	13	13	13	13	13	13	13	13
S2	11	4	6	6	8	8	8	8	8	9	9	9	9	9	9	9
S3	5	5	5	5	5	5	5	5	5	5	5	5	5	5	5	5
S4	36	4	8	14	17	20	24	27	29	30	31	32	32	33	33	35
S5	19	8	12	15	18	19	19	19	19	19	19	19	19	19	19	19
S6	6	4	5	5	5	5	5	5	5	5	5	5	5	6	6	6
S7	79	9	17	26	36	45	52	58	61	62	68	68	70	72	74	74
S8	5	3	4	4	4	4	4	4	4	4	4	4	5	5	5	5
S9	2	2	2	2	2	2	2	2	2	2	2	2	2	2	2	2
S10	12	3	10	11	11	12	12	12	12	12	12	12	12	12	12	12
S11	7	1	2	2	2	2	2	2	2	2	2	2	2	2	2	2
S12	7	3	4	5	6	6	6	7	7	7	7	7	7	7	7	7
S13	19	10	17	19	19	19	19	19	19	19	19	19	19	19	19	19
S14	14	9	12	12	12	13	14	14	14	14	14	14	14	14	14	14
S15	6	5	5	5	6	6	6	6	6	6	6	6	6	6	6	6
TOTAL	241	78	120	144	164	179	191	201	206	209	216	217	220	224	226	228
MEAN		32%	50%	60%	68%	74%	79%	83%	85%	87%	90%	90%	91%	93%	94%	95%

Nomination certainties are updated after every iteration, thereby allowing nominations with higher likelihoods to be shown first. Template reliabilities were also updated at the end of each review. This was done by updating the true and false positives of contributing templates and recalculating their reliability scores accordingly, as shown in Appendix S1.

**Figure 5:**
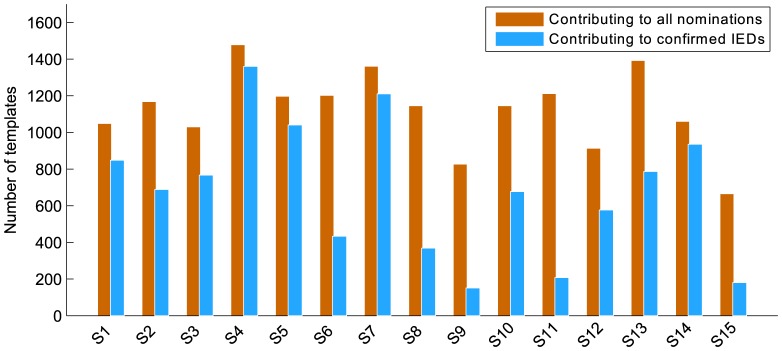
Template contributions per recording: The first bar shows the number of templates that have contributed to all the nominations presented during a review of fifteen iterations. The second bar shows the number of templates that contributed to confirmed IEDs only.

Figure 5 provides some insight into the number of templates involved during detection. A bar graph is shown consisting of two bars per subject. The first bar shows the number of templates contributing to all nominations presented during the review of fifteen iterations. The second bar shows the number of templates that contributed to confirmed IEDs. From this we see that a substantial number of templates are involved during detection, and more importantly, that many of them contribute to confirmed IEDs. We also see that many templates are responsible for negative contributions. It should be noted however that fifteen iterations were used per review, and that in most recordings, only a small number of inter-ictal events were present. This means that if all IEDs were confirmed, the system would only have false positives left to show. This figure also confirms that a sufficient number of template waveforms were available in the database to detect IEDs in the new, unseen EEGs of our evaluation dataset. Template reliabilities are not reflected in this graph, meaning that detections with high certainty are not separated from those with low certainty. Given the average reviewing time to perform one iteration, a typical review of fifteen iterations will approximately last five minutes for a 20–30 min EEG recording. This estimate is however only based on internal evaluation, and a larger study with multiple participants is required to obtain an accurate measure. The review time will be reduced even further if a reviewer is already satisfied with the nominations seen after fewer iterations.

## Discussion

A large number of inter-ictal spike detection methods have been proposed in recent years (see [1], [2]). Many of these have been designed with patient specific features, or have been tested on segmented EEG data with possibly reduced movement and eyeblink artifacts. For these reasons alone, they already appear not to scale well to new, unseen EEG recordings as we would wish for practical consideration. Given the nature of spike detection algorithms, it is also difficult to achieve both a high sensitivity and a low false detection rate simultaneously. Some studies therefore place their focus on achieving high sensitivities [8], [10], [17], [18], [20], whereas others aim to maintain low false positive rates [11], [13], [14]. In a practical scenario, this means that reviewers will be forced to look at a large number false positive events, or to see fewer nominations and miss out on important detections. When only these two options are available, it is clear to see why epileptologists prefer the more conventional method of visual inspection with manual detection. Our system has been optimized to achieve high sensitivities with events being presented to the reviewer in a minimal amount of time. A weakness in the system therefore lies in a higher number of false positives which range from 0.24–6.6 fp/min depending on the number of iterations chosen. Sensitivities for other reported methods range from 0.5 to 0.92 and false detection rates from 0.1 to 6 fp/min [1], [2].

This study presents an alternative approach to inter-ictal spike detection and review. It has three fundamental differences to other automated methods, which to our knowledge has not been used before: First, individual IED nominations are assigned a detection certainty, giving the system an opportunity to present them in a descending order of likelihood. Secondly, the described system can learn from the outcome of events that have been presented, and in such a way attempt to increase its sensitivity during presentation of the remaining nominations after every iteration. Lastly, the system can adapt itself on a continuous and universal basis, thereby improving its own accuracy over time. By storing the number of true and false positive detections of each template and updating their reliabilities accordingly, the template reliabilities will become more accurate which in turn will improve the overall robustness of the system.

Given that IED nominations are presented in a descending order of likelihood, the reviewer is not required to view all nominations before knowing if any inter-ictal activity exists. Instead, it is left up to the reviewer to decide on the number of nominations to view before a diagnosis is made. Because of this, the system is less vulnerable to high false positive rates. This approach also avoids the need for thresholding to limit the number of nominations presented to the reviewer, and should make it scalable to long-term recordings. Further evaluation is required to verify this.

Some methods try to overcome the burden on visual confirmation by clustering nominations together into groups based on similarity in space and morphology [7], [8], [20]. Few studies have given detailed descriptions on how detected IEDs are presented to reviewers, but the most similar technique to ours is given by [7]. In their method, the authors describe a system that uses clustering to group events based on a similarity measure calculated from a subset of 18 quantitative features. Their system orders the clusters by frequency of occurrence and presents the reviewer with one cluster at a time to review. The reviewer can confirm which clusters contain IEDs, and by confirming a cluster, all events contained within it are labeled as IEDs. Although this technique is similar to ours in some ways, a number of key differences exist. First, given that IEDs are confirmed in clusters and not as single events, the cluster-based review approach is vulnerable to confirming false detections clustered together with true IEDs. Although in our method the confirmation of single events will potentially take longer than confirming clustered events, the reviewer is assured that all marked IEDs will have been shown and are in fact true inter-ictal events. The second key difference is that, apart from ordering the clusters by frequency of occurrence, the cluster-based system does not have any certainty measure similar to the one we describe. All clusters therefore need reviewing in order not to miss important events, and this can result in longer review times. Lastly, our system is unique in incorporating reviewer feedback from current reviews to improve the detections on future recordings.

As with any method, a number of improvements can still be made to further increase the efficiency and accuracy of our technique. Specific areas of improvement include a built-in mechanism to collect and train additional templates for expanding the template database with more example waveforms. This will result in a completely closed-loop system, where better templates can be promoted over time and unreliable templates discarded. Additionally, context-based rules such as those described by [7], [8], [11], [17], [20] can be added to group nominations according to their similarity, which can be used to provide additional information during the review. Given that only 15 EEGs were used to evaluate this method and that reviewer bias may have been added by having the IEDs marked by only one reviewer, further evaluation is needed to determine the feasibility of this method in clinical practice. Further work will involve multiple electroencephalographers and a comparison between visual searching and automated detection in long-term recordings.

In summary, by adding information to detected events in the form of certainty values, nominated IEDs can be shown in a decreasing order of likelihood and thereby make it possible for the reviewer to view fewer events. In practical terms, this has the potential to improve efficiency and lower EEG review times, and thereby make automated detection faster than conventional manual reviews while achieving a similar diagnostic certainty.

## Supporting Information

Appendix S1 **Calculation of the group certainties.**
(PDF)
