# Supplementary material for: A Self-Adapting System for the Automated Detection of Inter-Ictal Epileptiform Discharges
Source: PLoS One. 2014 Jan 15;9(1):e85180. doi: 10.1371/journal.pone.0085180 (PMC3893182; doi:10.1371/journal.pone.0085180)
Supplement: Appendix S1 — Calculation of the group certainties. (PDF) [file pone.0085180.s001.pdf]

Appendix S1

Calculation of the group certainties

Using the same method as described in [16], a database of stored template waveforms make individual detections of inter-ictal activity by finding events in the EEG that have high correlations to themselves. Individual detections are merged and grouped where they overlap, and from this the grouped IED nominations are formed (see illustration Fig. 3).

Let each individual template detection be defined as

$$D_{tem} = \{CRR_{tem}; TP_{tem}; FP_{tem}; R_{tem}\}, \tag{1}$$

where CRR_{tem} is the correlation coefficient between the template and the detected segment, TP_{tem} and FP_{tem} the total number of true and false detections made by that template over all time (stored in the database), and R_{tem} a measure of the template reliability:

$$R_{tem} = \frac{TP_{tem}}{TP_{tem} + FP_{tem}}. \tag{2}$$

Given that each IED nomination can be seen as a combination of N overlapping detections ($nom = \{D_{tem}\}, \ tem \in [1...N]$), the certainty value of that nomination is calculated as

$$CERTAINTY_{nom} = \max\left(\{CRR_{tem} \times R_{tem}\}\right), \quad tem \in nom. \tag{3}$$
